# The Cognitive Association Between Effortful Self-Control and Decreased Vitality

**DOI:** 10.3389/fpsyg.2021.631914

**Published:** 2021-04-28

**Authors:** Alex Bertrams

**Affiliations:** Educational Psychology Lab, Institute of Educational Science, University of Bern, Bern, Switzerland

**Keywords:** ego depletion, effort, energy, fatigue, schema model of self-control, self-control, self-regulation, subjective vitality

## Abstract

According to the schema model of self-control, individuals’ self-control efforts activate the fatigue/decreased vitality schema. A precondition for this schema activation is that the cognitive concepts of self-control effort and decreased vitality are associated in individuals’ minds. In the present two studies, the existence of such a cognitive association was tested. In Study 1, 133 school students from Switzerland read two similar stories in a random order. In one story, a fictitious individual engaged in effortful self-control, while in the other story, he/she did not. In Study 2, 251 online workers from the United States, per random assignment, received either a story describing an individual exerting self-control or a similar story describing an individual not exerting self-control. In both studies, the participants rated how vital the fictitious individuals felt at the time the story ended. As expected, in both studies, the fictitious individual exerting self-control was rated as feeling less vital compared to the one not exerting self-control. This finding is in line with the schema model of self-control, as it indicates that the concepts of self-control exertion and decreased vitality are related to each other in a cognitive associative structure. Additional results suggest that emotional valence and calmness are irrelevant in this association. Moreover, the self-control exertion-decreased vitality association was independent from the raters’ own momentary feelings of self-control exertion, effort, and exhaustion.

## Introduction

*Self-control* is the overriding or altering of a predominant response tendency (Baumeister et al., 2007). It is required when easily available intuitive or automatic responses would not help or even counteract the achievement of goals. For instance, the habit of checking one’s social media updates may be one’s predominant response, experienced as immediately gratifying but not useful for achieving a satisfying grade on a math exam. Therefore, replacing this predominant response with studying for the exam would constitute self-control in the service of a higher aim (Duckworth et al., 2019). Previous research has demonstrated that being good at self-control positively contributes to individuals’ performance in cognitive and perceptual-motor tasks (Englert and Bertrams, 2012; Bertrams et al., 2013), success in the educational system (Duckworth et al., 2019), social relationship quality (Vohs et al., 2011), good health (Crescioni et al., 2011), well-being (Vötter and Schnell, 2019), and ethical behavior in the workplace (Li et al., 2020; Ming et al., 2020). Overall, being good at self-control appears to be key to success in life (Baumeister et al., 1998). Therefore, it is an interesting psychological phenomenon that individuals fail in self-control from time to time, that is, there are intra-individual fluctuations in individuals’ self-control capacities (Baumeister and Vohs, 2016).

There are several theories explaining failure at self-control. The most prominent may be the *strength model of self-control* (Baumeister et al., 1994; Baumeister and Vohs, 2016), which set the stage for research in modern empirical psychology as to when and why individuals fail at self-control. According to the strength model, successful self-control depends on a limited resource, akin to strength that is taxed by exerting self-control. After the initial exertion of self-control, the resource is temporarily depleted (a state called *ego depletion*), and, therefore, subsequent self-control is impaired (i.e., the *ego depletion effect*). The strength model has one crucial gap: the nature of the limited resource; thus, the process underlying the self-control resource depletion is still unknown. Alternative theories that have followed the strength model have other explanatory problems (Bertrams, 2020). Therefore, Bertrams (2020) recently developed the *schema model of self-control*, which is principally compatible with the strength model of self-control but is more precise in defining the ego depletion process that decreases self-control after initial self-control demands.

**Figure 1:**
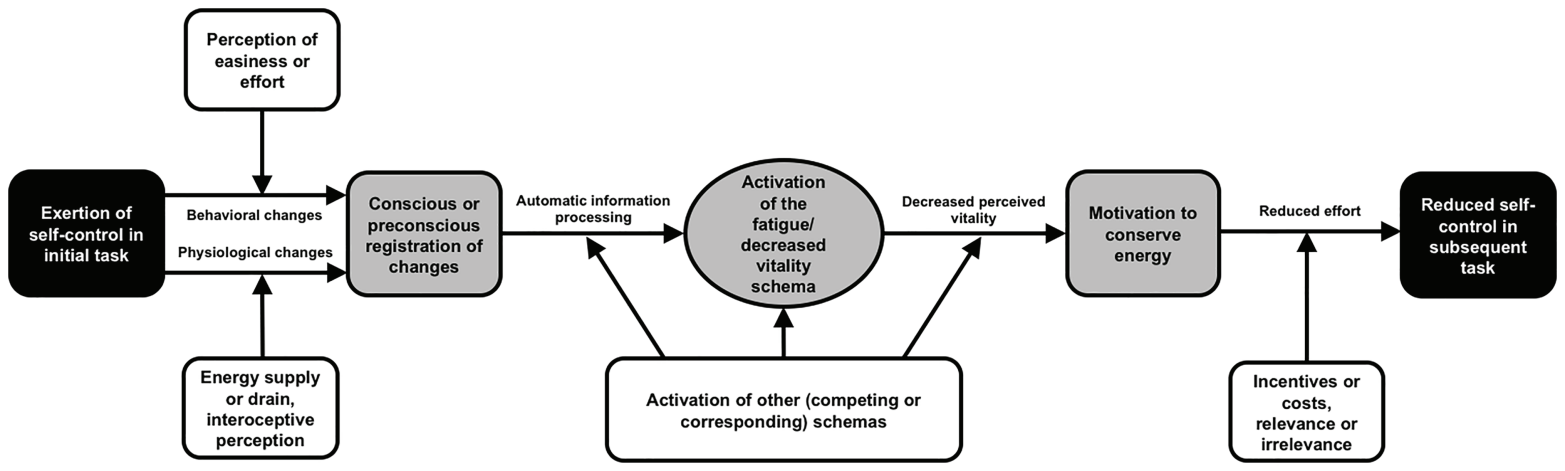
The schema model of self-control (Figure taken from Bertrams, 2020). Black boxes: the observable behavior in self-control studies. Gray boxes and horizontal arrows: the mediating processes within the individual. White boxes: moderating variables.

Simply put, according to the schema model, the exertion of effortful self-control is accompanied by the registration of behavioral and physiological changes that activate the fatigue/decreased vitality schema (Figure 1, left half). Individuals who exert self-control may experience that their own behavior has altered from before the ongoing self-control demand to the present moment toward a behavior that feels more effortful. The thereby activated schema then causes a decrease in their perceived vitality (i.e., the energy one feels he/she momentarily possesses for the regulatory control of him-/herself; Ryan and Frederick, 1997). Consequently, one becomes motivated to conserve energy and reduce effort, and, hence, further self-control declines (Figure 1, right half). At several positions within the model, moderators are postulated that can affect the process (Figure 1, white boxes). While the process depicted in Figure 1 can be preconscious, Bertrams (2020, 2021) assumed that some parts can come to consciousness, particularly when the intensity of the self-control demand involved is high. In the logic of the schema model, it is not primarily important whether a limited self-control resource exists (although its existence is considered possible). The decisive factor for decrements in self-control to occur is that the fatigue/decreased vitality schema is activated. This activation can be initiated by a real or imagined energy loss. So, at its core, the schema model states that individuals act at least *as if* their self-control depends on a limited resource.

As Bertrams (2020) pointed out, to evaluate the usefulness of the schema model of self-control, further research is needed to support the existence of the fatigue/decreased vitality schema. There is already research that highlights the crucial mediating role of perceived fatigue in the ego depletion process (Graham et al., 2017). However, one central aspect that has yet to be shown is that effortful self-control behavior is *mentally* associated with fatigue or decreased vitality (note that within the framework of the schema model, decreased vitality is considered equivalent to increased fatigue; Bertrams, 2020). This association is integrated in the left half of Figure 1, where behavioral changes (from less to more effortful behavior) are registered and processed, and activate the fatigue/decreased vitality schema. Thus, for schema activation, even the mental concept of self-control effort has to be causally related to decreased vitality. This cognitive if-then association is required for translating actual self-control behavior into the assumed information-processing mechanism (Figure 1). The present two studies were designed to investigate this schematic association in a school student sample from the German-speaking part of Switzerland (Study 1) and in a diverse online sample of United States residents (Study 2).

In Study 1, the participants successively read two stories in a random order in which the same fictitious character on one day exerted effortful self-control but did not the other (within-subjects design). In Study 2, all participants first read a story about a character who did not exert self-control. Afterward, they read a story about a character who, per random assignment, had either exerted effortful self-control or not (mixed between-within subject design). After each story, the participants rated the character’s perceived availability of energy (i.e., perceived vitality). Similar procedures have been used in the past to show schematic associations, for example, between the concepts of dryness and vitality (Shalev, 2014). Analogous to Shalev (2014, Study 4a and Study 4b), the participants rated the experiences of fictitious individuals. In this way, conclusions about a generally existing cognitive association between self-control effort and decreased vitality could be made (i.e., independent from individual features that the participants perceived regarding themselves or their familiar ones). It was predicted that the characters in the stories would be rated as less vital in the case where they just exerted self-control compared to the case where they did not.

## Study 1

The participants rated a fictitious individual’s experiences, including his/her perceived vitality, twice – in one situation with self-control and in another situation without self-control. In addition to the central comparison of the perceived vitality between the two self-control conditions, relational patterns between the ratings of perceived vitality, effort, amount of self-control exerted, positive valence, and calmness were examined. These supplementary analyses were intended to elucidate the crucial role of decreased perceived vitality as a mental concept that is schematically associated with the exertion of self-control.

### Methods

#### Participants

The final sample comprised 133 participants (52%/48% female/male; *M*_age_ = 19.14, *SD*_age_ = 7.28) who were recruited from different schools in the German-speaking part of Switzerland. Data on 13 additional individuals were not analyzed due to its incompleteness. A power analysis using G∗Power 3.1 (Faul et al., 2007) indicated that a total sample size of at least 100 participants would be required to detect a medium-size effect with high statistical power (power analysis: *t*-test, matched pairs, two-tailed; input parameters: *d*_z_ = 0.50, *α*_Bonferroni corrected_ = 0.01, 1−*β* = 0.99). A medium-size effect was assumed as default (cf. Wolff et al., 2019) since there has been no previous study on this question. Given its sample size, the present study was well-powered.

#### Procedure and Measures

The participants were informed that the present study was about the imagination of other individuals’ inner states. After providing socio-demographic data, the participants read two stories (see the Supplementary Material to this article). The order in which the participants read the two stories was counterbalanced *via* random assignment. In one story (self-control condition), the participants were asked to imagine a fictitious character employed at an office who was clearly right-handed and not at all used to using his/her left hand for everyday activities typically performed with his/her right hand. It was further illustrated that the fictitious character had made a bet to perform his/her routine for an entire normal working day like a left-handed individual (e.g., cutting and buttering bread, making coffee, brushing teeth, combing hair, buttoning clothes, unlocking and locking doors, using the computer mouse, writing notes on paper, using a knife at lunch, writing on a flipchart, etc.). Then, the participants were asked to take a moment to reflect on and imagine the ongoing activities performed with the left hand from the perspective of the right-handed fictitious character. Using one’s non-dominant hand requires overriding habitual behaviors and exerting control over one’s attention and movements; therefore, it represents a form of self-control (Baumeister and Vohs, 2016). The other story (no-self-control condition) was like the story in the self-control condition, except that the participants were asked to imagine a normal working day during which the same fictitious character used his/her dominant right hand as usual. Again, the participants were instructed to reflect on and imagine the activities with the right hand from the perspective of the right-handed fictitious character. Importantly, none of the internal experiences of the fictitious character, such as effort, fatigue, mood, vitality, nor any words directly referring to self-control, were mentioned in the stories.

After each story, the participants completed the same series of items on six-point scales with named poles. For all items, the time between the fictitious character’s waking up in the morning and 4:30 pm of the said day was given as the temporal frame of reference. First, two items assessed the fictitious character’s experienced effort from the participants’ perspective (*effortless* [1] to *strenuous* [6]; *difficult* [1] to *easy* [6]; prior to averaging the responses to the two items, the second item was recoded). The effort items were designed similarly to manipulation checks in prior studies on self-control strength (e.g., Bertrams and Englert, 2019; Kühl and Bertrams, 2019). Then, the participants rated two items as to how much the fictitious character had controlled his/her own behavior (*did not control own behavior at all* [1] to *controlled own behavior very much* [6]; *did not overcome habits at all* [1] to *overcame habits very much* [6]; the two items were averaged). Afterward, the participants completed a validated six-item mood measure (Wilhelm and Schoebi, 2007) to rate the fictitious character’s feelings. This measure comprised two items for each of the three subscales: *valence* (*content* [1] to *discontent* [6], recoded; *unwell* [1] to *well* [6]), *calmness* (*agitated* [1] to *calm* [6]; *relaxed* [1] to *tense* [6], recoded), and *vitality* (*tired* [1] to *awake* [6]; *full of energy* [1] to *no energy* [6], recoded). For each subscale, the items were averaged after the recoding. The subscale *vitality* was the measure of central interest in the present study. Finally, the participants were thanked and debriefed.

### Results and Discussion

#### Mean Comparisons

For the within-subjects comparisons, five paired sample *t*-tests with two-tailed testing and a Bonferroni correction were applied. As shown in Table 1, the participants rated the fictitious character’s experienced effort and exerted self-control as higher under the self-control condition than under the no-self-control condition. This indicates that the self-control manipulation was successful. Moreover, the participants estimated positive valence and calmness to be lower under the self-control condition than under the no self-control condition. Thus, self-control exertion seemed to be stored in the participants’ memories as an unpleasant and tense experience.

**Table 1:** Descriptive statistics and mean comparisons between the ratings in the self-control condition and no-self-control condition in Study 1.

Rating of the fictitious individual	Self-control condition		No-self-control condition		Paired samples *t*-tests		
	*r*_items_	*M* (*SD*)	*r*_items_	*M* (*SD*)	*t*(132)	*p*	*d*_z_
Effort experienced	0.53	4.81 (1.10)	0.49	1.68 (1.05)	19.67	<0.001	1.71
Self-control exerted	0.40	4.45 (1.32)	0.46	2.51 (1.41)	9.41	<0.001	0.82
Perceived positive valence	0.52	3.36 (1.27)	0.51	4.63 (1.02)	−8.80	<0.001	−0.76
Perceived calmness	0.42	3.01 (1.15)	0.52	4.52 (1.08)	−10.95	<0.001	−0.95
Perceived vitality	0.35	2.89 (1.14)	0.45	3.88 (1.09)	−7.18	<0.001	−0.62

*N* = 133. *r*_items_ = correlation between the two items of a measure (all *p* < 0.001).

Most importantly, the fictitious character’s perceived vitality was rated lower when the participants imagined the fictitious character exerting self-control instead of not exerting self-control. The interpretation can be that there is a cognitive association between the mental concepts of effortful self-control and decreased vitality, as proposed by the schema model of self-control. All values of *p* were <0.001, and all effect sizes were medium to large according to Cohen’s (1988) classification (medium effect: |d| ≥ 0.50; large effect: |d| ≥ 0.80).

#### Supplementary Analyses

In the no-self-control condition, the rating of perceived vitality was substantially related to the positive valence rating (*r* = 0.47, *p* < 0.001) and the calmness rating (*r* = 0.43, *p* < 0.001). In contrast, in the self-control condition, the rating of perceived vitality was unrelated to the rating of positive valence (*r* = 0.07, *p* = 0.41) and only slightly correlated with the rating of calmness (*r* = 0.17, *p* = 0.05). The positive valence and calmness ratings were highly correlated under the no-self-control condition (*r* = 0.70, *p* < 0.001) and the self-control condition (*r* = 0.71, *p* < 0.001). When the calmness rating under the self-control condition was entered as a covariate in a repeated measures analysis of covariance, the rated perceived vitality was still lower under the self-control condition than under the no-self-control condition (*F*[1, 131] = 10.86, *p* = 0.001, *η*^2^_partial_ = 0.08). In summary, without the context of exerting self-control, the participants’ concept of perceived vitality was apparently part of a diffuse affective experience. In the context of the imagined exertion of self-control, perceived vitality seemed to be a concept separate from emotional valence and calmness.

**Figure 2:**
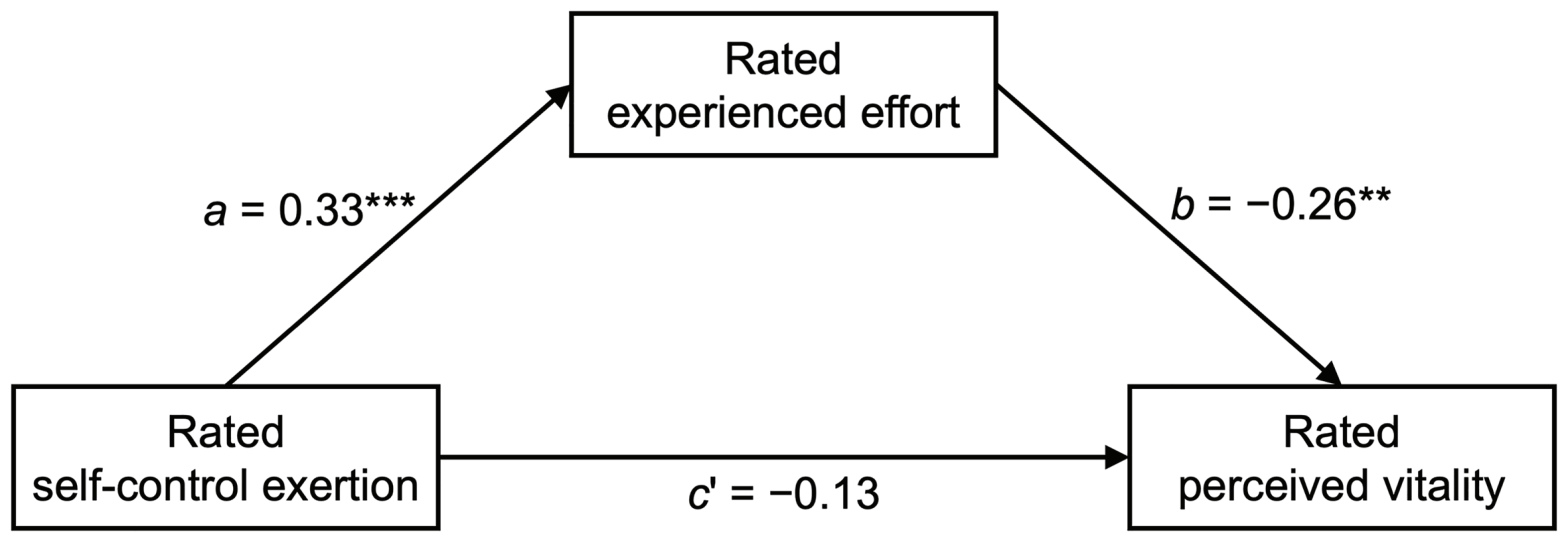
Mediation of the direct effect (*c*’) of rated self-control exertion on rated perceived vitality *via* the mediator rated experienced effort in the self-control condition (Study 1). Depicted are the standardized beta weights. *N* = 133. ^*^p < 0.05; ^**^p < 0.01; ^***^p < 0.001.

Under the self-control condition, the rated perceived vitality was negatively correlated with the rated experienced effort (*r* = −0.30, *p* < 0.001) and rated exertion of self-control (*r* = −0.22, *p* = 0.01). A mediation analysis (Figure 2) using the regression-based tool PROCESS (Hayes, 2013) revealed that the rated experienced effort mediated the negative relationship between the rated exertion of self-control and the rated perceived vitality under the self-control condition (*ab*_indirect effect_ = −0.07, 95% CI_indirect effect_ [−0.15, −0.03]; bias-corrected, bootstrapping with 5,000 resamples). This pattern conforms to the assumption of the schema model of self-control that the mental registration of an increase in behavioral effort during self-control activates the fatigue/decreased vitality schema.

The positive valence rating was unrelated to the experienced effort rating under the self-control condition (*r* = −0.17, *p* = 0.06), whereas the rated calmness and effort were correlated (*r* = −0.20, *p* = 0.03). Both the rated positive valence (*r* = 0.10, *p* = 0.28) and the rated calmness (*r* = −0.03, *p* = 0.70) were unrelated to the rated self-control exertion under the self-control condition. Thus, only perceived vitality had a consistent association with both effort and exertion of self-control, underlining its special role in the context of self-control.

## Study 2

The second study was intended to demonstrate the cognitive association between effortful self-control and decreased vitality once more – this time in a diverse online sample of United States residents. In this case, the perceived causality in this relationship was examined more closely by applying an experimental between-within design. Another aim was to show that the expected pattern would emerge independent of the raters’ own experiences of effort, self-control, and exhaustion during the experiment. This way, the possibility that effortful self-control and decreased vitality would just be associated as a temporal illusory correlation driven by momentarily easily available information should be ruled out. Thus, further evidence was sought supporting a general, temporally stable schematic association between effortful self-control and decreased vitality. In contrast to Study 1, the stories were less basic this time; that is, they were more narrative in style and the protagonists were given names and genders. This was done to establish a stronger reference to reality.

As a baseline measure, all participants first read a story about a character who did not exert extra self-control and were asked to rate this character’s perceived vitality. After that, the participants read a story about a character who, per random assignment, either exerted extra self-control or did not. The participants in both experimental conditions rated the perceived vitality of the character in the second story.

### Methods

#### Participants

The final sample comprised 251 participants from the United States (40%/60% female/male; *M*_age_ = 37.43, *SD*_age_ = 10.80) who were recruited from Amazon’s Mechanical Turk platform. Regarding educational background, 34 participants had completed high school or less, nine had completed 12 years of education plus non-academic training, 70 had completed 13–14 years of education plus some college or an associate degree, 111 had completed college with a bachelor’s degree, and 27 participants had completed academic education beyond a bachelor’s degree. Prior to the analyses, it was decided not to analyze the data of 50 individuals because they failed the attention check. However, the results did not change when these data were included in the auxiliary analyses. The data of three other individuals were incomplete and not analyzed. The participants received US $1.50 for their participation.

A power analysis (G∗Power 3.1; Faul et al., 2007) showed that a total sample size of at least 150 participants would be required to detect an at least medium-size effect with high statistical power, even in the unfavorable case that the repeated measures would not be correlated (power analysis: analysis of variance, repeated measures, within-between interaction; input parameters: *f* = 0.25, *α* = 0.05, 1−*β* = 0.99; number of groups = 2; number of measurements = 2; correlation_repeated measures_ = 0; *ε* = 1). Twice the number of individuals were recruited to buffer against a barely predictable number of individuals whose data had to be excluded (failed attention check). With *N* = 251, the present study was well-powered.

#### Procedure and Measures

Potential participants were invited on the online platform to participate in research on how people comprehend stories and evaluate characters in stories upon taking the protagonist’s perspective. First, the participants completed an attention check. For this, a task from previous research (Bertrams and Schlegel, 2020) was used to check whether the participants carefully read and followed the instructions. The question “Who was the first president of the United States of America?” was followed by instructions not to check any of the three response options but to click “continue.” Data from the participants who did not correctly follow this instruction were excluded from the analyses.

After providing their socio-demographic data, the participants were informed that they would read two stories and were instructed to take the perspective of the protagonists, that is, to really imagine the behaviors, thoughts, and feelings of each character in the stories and mentally simulate their actions (the stories are provided in the Supplementary Material to this article). Next, all participants read the same story about Chris, a professor who made his/her way from home to a university in the morning and followed his/her usual routine (i.e., no extra self-control). Pronouns were automatically adapted to the gender of each participant to facilitate the perspective-taking. No references were made to how Chris felt (e.g., perceived effort, vitality). Afterward, the participants completed the same measure of perceived vitality as in Study 1 regarding how Chris would feel in the situation in which the story ended.

Then, per random assignment, the participants read one of two other stories. In the self-control condition (*n* = 132), it was described how Sam, a right-handed accountant, performed all activities with his/her left-hand on International Lefthanders Day. The story in the no-self-control condition (*n* = 119) was similar to the story in the self-control condition, except Sam was left-handed and performed everything as usual on International Lefthanders Day. Again, pronouns were adapted to each participant’s gender. None of the studies mentioned how Sam felt (e.g., perceived effort, vitality). After reading the respective story, the participants completed the measure of perceived vitality (see Study 1) a second time. The participants rated how Sam would feel during the afternoon of International Lefthanders Day.

Subsequently, the participants completed a three-item manipulation check as to how much the protagonist in the second story exerted effortful self-control (“To what extent did Sam need to control himself/herself?”; “To what extent did Sam need to concentrate during the day?”; and “To what extent did Sam have to go against his/her instincts?”). Each item was answered on a six-point response scale from 1 (*not at all*) to 6 (*very much*). The responses were averaged across the three items (Cronbach’s *α* = 0.94). Furthermore, on three items, the participants indicated their own effort (“How difficult was the task?”), self-control exertion (“How much did you have to concentrate during the task?”), and exhaustion (“How exhausting was the task?”), each on six-point scales from 1 (*not at all*) to 6 (*very much*). Finally, the participants were thanked, debriefed, and paid.

### Results and Discussion

#### Manipulation Check

The participants rated the protagonist’s (Sam) self-control exerted under the self-control condition (*M* = 5.40, *SD* = 0.75) as being higher than under the no-self-control condition (*M* = 2.43, *SD* = 1.43; *t*[249] = 20.86, *p* < 0.001, *d* = 2.64), indicating that the manipulation was successful.

#### Mean Comparisons

A 2 (experimental condition: self-control condition vs. no-self-control condition) × 2 (repeated measure: first story vs. second story) mixed between-within subject analysis of variance with perceived vitality as the dependent variable was conducted. There were significant main effects of the between-factor (experimental condition; *F*[1, 249] = 31.04, *p* < 0.001, *η*^2^_partial_ = 0.11) and the within-factor (repeated measures; *F*[1, 249] = 55.35, *p* < 0.001, *η*^2^_partial_ = 0.18). Most importantly, the interaction effect was also significant as expected (*F*[1, 249] = 30.84, *p* < 0.001, *η*^2^_partial_ = 0.11). To interpret the interaction effect, the means (Table 2) were compared using a row of *t*-tests (Bonferroni adjusted significance level: *α* = 0.0125). In the self-control condition, perceived vitality in the second story was rated lower than in the first story (baseline without self-control; *t*[131] = 8.20, *p* < 0.001, *d*_z_ = 0.71). Perceived vitality in the second story was also rated lower in the self-control condition than in the no-self-control condition (*t*[249] = 7.30, *p* < 0.001, *d* = 0.92). The two other mean comparisons were nonsignificant (*p* > 0.10). All the results described in this paragraph did not change when the participants’ own effort, self-control exertion, and exhaustion were controlled as covariates. In summary, the findings indicate that the effortful exertion of self-control is conceptualized in individuals’ minds as one cause of decreased perceived vitality.

**Table 2:** Descriptive statistics for the perceived vitality ratings in Study 2.

Story	Self-control condition (*n* = 132)		No-self-control condition (*n* = 119)	
	*r*_items_	*M* (*SD*)	*r*_items_	*M* (*SD*)
1 (Baseline: no self-control in both conditions)	0.67	4.67 (1.06)	0.79	4.72 (1.17)
2 (Manipulation: self-control vs. no self-control)	0.74	3.31 (1.42)	0.74	4.53 (1.18)

*N* = 251. *r*_items_ = correlation between the two perceived vitality items (all *p* < 0.001).

## General Discussion

In two studies with different samples, the perceived vitality of fictitious characters was rated lower for situations involving extra self-control effort relative to situations without an additional self-control demand. This finding indicates that individuals possess a cognitive association between the mental concepts of effortful self-control and decreased vitality, consistent with the schema model of self-control (Bertrams, 2020). A crucial distinction between this finding and the previous finding of Muraven et al. (2008) has to be made. Muraven et al. showed that individuals who had actually exerted self-control subsequently felt less vital than individuals who did not engage in self-control. Their results could have been caused either by a cognitive association between the exertion of self-control and decreased vitality or by a real loss of any form of energy or both. In contrast, the present results are only explainable by a cognitive self-control exertion-decreased vitality association, as the participants themselves did not engage in actual self-control. Therefore, the present two studies were a focused attempt to falsify the schema model of self-control, which explicitly posits this cognitive association.

To further support the schema model, in Study 1, the participants’ ratings of the character’s experienced effort mediated the relationship between the ratings of self-control exertion and decreased perceived vitality (Figure 2). This relational pattern reflects a crucial part of the overall process within the schema model, namely, the activation of the fatigue/decreased vitality schema by registering an increase in behavioral effort (left half of Figure 1). Thus, for schema activation, it may not be sufficient to exert self-control but the individual must also perceive the self-control as strenuous. If there is reason to experience a specific self-controlled behavior as requiring no effort (by priming individuals in this direction or distracting them from the perception of effort), the fatigue/decreased vitality schema should not be activated (see the respective possibility of moderation in the left half of Figure 1). It should also be emphasized that the findings from the present mediation analysis revealed nothing about the second possible pathway (i.e., registration of physiological changes), which, following the schema model, can activate the fatigue/decreased vitality schema as well.

As a further result, in the context of self-control exertion, decreases in positive valence and calmness could be differentiated from decreases in perceived vitality and were not consistently associated with ratings of self-control exertion and effort. In contrast, the fictitious character’s rated perceived vitality was related to his/her rated self-control exertion and effort. This relational pattern points to the specific role of perceived vitality in the mental associative structure around self-control exertion. It is also mentionable that the identified mental self-control exertion-decreased vitality association was independent of the raters’ momentary self-control exertion, effort, and feelings of exhaustion. Therefore, this cognitive association seems to be generally stored in the memory and not a judgment mainly based on the raters’ momentary behaviors and inner states.

The present findings agree with the schema model of self-control. However, they are limited to a specific part of the model and do not confirm the model as a whole. The origin of the cognitive relationship between self-control exertion and decreased perceived vitality is also an open question. Possibly, due to evolutionary mechanisms, people are predisposed to easily develop such an association (Bertrams, 2020). Another limitation is that, in the present two studies, only one specific form of self-control (i.e., deliberate motor control) was described in the stories applied. Beyond self-control, this kind of behavior is also likely to be associated with other characteristics that people may consider energy consuming (i.e., enhanced expenditure of physical effort and time when using the non-dominant hand). The mediation analysis in Study 1 suggests that there is actually a mental association between the concepts of self-control exertion, effort, and vitality. Still, future research should use further types of self-control to extend the present findings and disentangle self-control from other efforts. Moreover, relevant moderators of the cognitive association between self-control exertion and decreased vitality should be examined. One crucial moderating influence may be whether an individual implicitly believes in the limited or unlimited availability of willpower (Job et al., 2010; Compagnoni et al., 2020). Following the schema model of self-control, it can be assumed that people who believe in the limitedness of willpower more strongly associate the exertion of self-control with reduced vitality than people who believe that willpower is an infinite capacity. In future research, the paradigm of this study can also be used to identify devitalizing self-control demands for specific groups of individuals (e.g., the compensatory efforts of autistic individuals; Bertrams and Schlegel, 2020).

## Data Availability Statement

The raw data supporting the conclusions of this article will be made available by the author, without undue reservation.

## Ethics Statement

The studies involving human participants were reviewed and approved by the Ethics Committee of the Faculty of Human Sciences at the University of Bern. The participants provided their written informed consent to participate in this study.

## Author Contributions

The author confirms being the sole contributor of this work and has approved it for publication.

### Conflict of Interest

The author declares that the research was conducted in the absence of any commercial or financial relationships that could be construed as a potential conflict of interest.
