# Supplementary material for: The Cognitive Association Between Effortful Self-Control and Decreased Vitality
Source: Front Psychol. 2021 Apr 28;12:631914. doi: 10.3389/fpsyg.2021.631914 (PMC8113642; doi:10.3389/fpsyg.2021.631914)
Supplement: Supplementary file 1 [file Data_Sheet_1.PDF]

frontiers

Supplementary Material

1 Stories Used in Study 1

1.1 Self-Control Condition (if First in Order)

Please imagine a completely fictitious person whom you do not know. This person is employed in an office. Imagine this fictitious person as clearly right-handed. This person is not at all accustomed to using the left hand for activities that he or she otherwise performs with the right hand in everyday life.

This person has made a bet and will now do everything for a whole Wednesday (a normal working day in the office), as if this person was left-handed. Among other things, the person will perform these activities—differently than usual—with the left hand: cutting and buttering bread, making coffee, brushing teeth, combing hair, buttoning clothes, unlocking and locking doors, using the computer mouse, writing notes on paper, using the knife at lunch, writing on a flip chart, etc.

Now, please take a moment and imagine the ongoing left-handed activities from the perspective of the clearly right-handed fictitious person.

1.2 No-Self-Control Condition (if Second in Order)

Now, please imagine the same Wednesday (working day) of the same fictitious person again. However, this time without the person performing activities in an unusual way with the left hand. Among other things, the person performs these activities—as usual—with the right hand: cutting and buttering bread, making coffee, brushing teeth, combing hair, buttoning clothes, unlocking and locking doors, using the computer mouse, writing notes on paper, using the knife at lunch, writing on a flip chart, etc.

Now, please take a moment and imagine the ongoing right-handed activities from the perspective of the clearly right-handed fictitious person.

1.3 No-Self-Control Condition (if First in Order)

Please imagine a completely fictitious person whom you do not know. This person is employed in an office. Imagine this fictitious person as clearly right-handed. This person is not at all accustomed to using the left hand for activities that he or she otherwise performs with the right hand in everyday life.

This person will spend an entire Wednesday (a normal workday in the office) doing everything the way this person is used to doing it. Among other things, the person performs these activities—as usual—with the right hand: cutting and buttering bread, making coffee, brushing teeth, combing hair, buttoning clothes, unlocking and locking doors, using the computer mouse, writing notes on paper, using the knife at lunch, writing on a flip chart, etc.

Now, please take a moment and imagine the ongoing right-handed activities from the perspective of the clearly right-handed fictitious person.

1.4 Self-Control Condition (if Second in Order)

Now, please imagine the same Wednesday (working day) of the same fictitious person again. However, this time, due to a bet, the person will do everything for an entire Wednesday (a normal working day in the office), as if this person was left-handed. Among other things, the person will perform these activities—differently than usual—with the left hand: cutting and buttering bread, making coffee, brushing teeth, combing hair, buttoning clothes, unlocking and locking doors, using the computer mouse, writing notes on paper, using the knife at lunch, writing on a flip chart, etc.

Now, please take a moment and imagine the ongoing left-handed activities from the perspective of the clearly right-handed fictitious person.

2 Stories Used in Study 2

2.1 First Story: No-Self-Control Baseline (if Participant was Female; Pronouns in the Version for Male Participants in Brackets)

Please take the perspective of the person in this story. Try to put yourself in the described situation and take Chris's perspective.

Chris is a professor of Physics. Since it is morning, she [he] puts on her [his] coat, picks up her [his] briefcase, and leaves the house. Like every morning when she [he] goes to work, she [he] takes the no. 53 bus to the city center where the university is located.

Two stops in front of the university, she [he] gets off the bus to walk the rest of her [his] way to work. During the remaining 15 minutes of her [his] trip to work, she [he] mentally prepares herself [himself] for the upcoming university lecture. While Chris is walking along the street, lost in thought, she [he] stops just before the university at a small kiosk. Chris talks to the woman operating the kiosk and buys the daily newspaper. She [He] then walks up the road to the main building. Chris likes routine. She [He] buys her [his] newspaper at the same kiosk every morning so that she [he] can read it during her [his] lunch break.

When Chris is about to enter the university building, one of her [his] students approaches her [him] and asks for an appointment to discuss his bachelor's thesis. Chris thinks about it briefly and offers to stop by her [his] office the day after tomorrow at 11 o'clock. Then, she [he] climbs the stairs to the lecture hall, enters the room and starts her [his] lecture for the waiting first-semester students.

2.2 Second Story: Self-Control Condition (if Participant was Female; Pronouns in the Version for Male Participants in Brackets)

Please take the perspective of the person in the story. As you read this story, try to put yourself in the described situation and take Sam's perspective.

As Sam, an accountant, buys the newspaper on her [his] way to work this morning, she [he] notices the headline, "Tomorrow: International Left-handed Day". So far, she [he] had not heard anything about this special day for left-handers.

On the bus to work, Sam meets her [his] workmate Tim. As a left-hander, Tim already knew about International Left-handed Day and shares some information about it with Sam, who is right-handed.

Tim tells her [him], for example, that only a small minority of the population are left-handed. They talk about this issue all the way to work, and when they arrive at the office, Tim asks his whole team to only use their left hands during tomorrow's International Left-handed Day.

Sam wants to follow Tim's suggestion, and the next morning, she [he] starts making this significant change by brushing her [his] teeth with her [his] left hand. She [He] has to take the toothbrush in her [his] left hand and brush her [his] teeth with it. It's very unusual for Sam and of course, takes longer. At the office, she [he] has to open the front door and her [his] laptop and drink her [his] cup of coffee with her [his] left hand. Furthermore, at lunch, Sam has to take the fork in her [his] left hand to eat the meal. When Sam's cell phone rings after lunch, she [he] instinctively wants to pick it up with her [his] right hand. Finally, she [he] notices what she [he] is doing, puts the phone in her [his] left hand, and takes the call. She [He] also responds to her [his] many WhatsApp messages by typing with her [his] left hand. It takes much longer than usual, because every time Sam has to act against her [his] instincts.

Throughout the day, Sam needs to concentrate on doing everything with her [his] left hand. She [He] is constantly made aware of these everyday automatic actions due to the conscious execution of her [his] tasks with her [his] non-dominant left hand.

By afternoon, half of International Left-handed Day is already over.

2.3 Second Story: No-Self-Control Condition (if Participant was Female; Pronouns in the Version for Male Participants in Brackets)

Please take the perspective of the person in the story. As you read this story, try to put yourself in the described situation and take Sam's perspective.

As Sam, an accountant, buys the newspaper on her [his] way to work this morning, she [he] notices the headline, "Tomorrow: International Left-handed Day". Sam is a left-hander, but she [he] did not realize that this special day would be celebrated tomorrow.

On the bus to work, Sam meets her [his] workmate Tim. As a left-hander, Sam knew about International Left-handed Day (even though she [he] had not been sure of the date) and shares some information about it with Tim, who is right-handed. She [He] tells Tim, for example, that only a small minority of the population are left-handed. They talk about this issue all the way to work, and when they arrive at the office, Tim asks his whole team to only use their left hands during tomorrow's International Left-handed Day. For many, it will be a significant change, but of course, there will be no change for Sam other than that she [he] will be a little more aware of what she [he] is doing with her [his] left hand.

The next morning, Sam's day starts with her [him] brushing her [his] teeth with her [his] left hand as usual. At the office, she [he] opens the front door and her [his] laptop with her [his] left hand and drinks a cup of coffee, also with her [his] left hand. At lunch, Sam takes the fork in her [his] left hand to eat her [his] meal. When Sam's cell phone rings after lunch, she [he] picks it up with her [his] left hand. She [He] also responds to her [his] many WhatsApp messages by typing with her [his] left hand. Throughout the day, Sam does everything instinctively with her [his] left hand as usual.

However, because of International Left-handed Day, Sam is constantly aware that she [he] is using her [his] left hand to carry out these everyday automatic actions due to her [his] conscious use of her [his] dominant left hand. For Sam, it is normal to do everything with her [his] left hand, and it is nothing special in the way it is for most of the right-handed people participating in the day.

By afternoon, half of International Left-handed Day is already over.
